# Dental College at Syracuse, N. Y.

**Published:** 1851-12

**Authors:** 


*Dental College at Syracuse, N. Y.* — A new college of dentistry, has recently been established at Syracuse, N. Y. Prof. Thomas Spencer, formerly of the Geneva and Chicago Medical Schools, and Prof. Shipman, late Prof. of Surgery at the Indiana Med. College, are members of the Faculty. From their experience in medical instruction, the services of these gentlemen will contribute to the success and usefulness of the institution with which they are at present connected.

